# Anti-PD-L1 monoclonal antibody for the management of chronic disseminated intravascular coagulation secondary to a urothelial carcinoma: a case report

**DOI:** 10.1186/s13256-022-03338-2

**Published:** 2022-03-21

**Authors:** Silvia Maiorano, Wiebke Gulden-Sala, Bernhard Gerber, Guido Ghilardi

**Affiliations:** 1 Department of Internal Medicine, Ospedale la Carità, EOC, Locarno, Switzerland; 2 Department of Hematology, Istituto Oncologico della Svizzera Italiana (IOSI), Bellinzona, Switzerland; 3 Department of Internal Medicine, Ospedale S. Giovanni, EOC, Bellinzona, Switzerland

**Keywords:** DIC, Thrombocytopenia, Atezolizumab, Urothelial carcinoma, Case report

## Abstract

**Background:**

Thrombocytopenia is often considered a risk factor for bleeding, but conversely may be associated with an increased thrombotic risk in several clinical situations. Here we present a patient with arterial thrombosis and chronic disseminated intravascular coagulation caused by metastatic urothelial carcinoma. As the treatment for a disseminated intravascular coagulation caused by a neoplasia is the treatment of the underlying disease itself, our case highlights a new therapeutic approach—immunotherapy—in a patient prone to hematological complications due to conventional chemotherapy.

**Clinical case:**

A 74-year-old Caucasian male patient with a history of urothelial carcinoma of the bladder and moderate thrombocytopenia had multiple arterial thrombotic events despite antiplatelet therapy and anticoagulation. A diagnosis of chronic disseminated intravascular coagulation in the setting of a metastatic bladder urothelial carcinoma was made. The patient was treated with an anti-PD-L1 monoclonal antibody, and achieved a rapid response with subsequent reversal of the disseminated intravascular coagulation.

**Conclusion:**

Unexplained arterial or venous thrombosis despite adequate thromboprophylaxis should be investigated, especially in the setting of thrombocytopenia. Chronic disseminated intravascular coagulation is a possible, life-threatening reason for this clinical picture, and should prompt rapid identification of the underlying disease. To the best of our knowledge, this is the second case of chronic disseminated intravascular coagulation due to neoplastic disease treated with immunotherapy.

## Background

Although thrombocytopenia is usually considered a risk factor for bleeding events, there are a number of conditions where thrombocytopenia is instead associated with thromboembolic events.

The most important disease entities leading to thrombotic complications despite underlying thrombocytopenia are heparin-induced thrombocytopenia (HIT) [1], antiphospholipid syndrome (APS) [2], disseminated intravascular coagulation (DIC) [3], thrombotic microangiopathy (TMA) [4], paroxysmal nocturnal hemoglobinuria (PNH) [5], and immune thrombocytopenia (ITP) [6] in some situations. The pathophysiological mechanism of thrombocytopenia and thrombosis is complex and depends on the biological characteristics of the underlying condition. One of these conditions is chronic DIC, which is an acquired and heterogeneous disorder characterized by the widespread activation of coagulation and fibrinolysis cascades, resulting in the intravascular formation of fibrin and, ultimately, thrombotic occlusion of small vessels [7]. A DIC score proposed by the International Society on Thrombosis and Haemostasis (ISTH) might help to diagnose and quantify the extent of DIC in patients with an underlying condition known to be associated with coagulation activation [8, 9]; a score of 5 or more points is considered diagnostic for DIC.

Here we present the case of a patient presenting with multiple thromboembolic events and persistence of thrombocytopenia.

## Case presentation

A 74-year-old Caucasian man was referred to the emergency department in May 2018, presenting with mild and reversible neurological symptoms. Magnetic resonance imaging (MRI) revealed multiple ischemic strokes. The patient was taking aspirin 100 mg daily as secondary prophylaxis for ischemic and valvular cardiopathy treated with percutaneous transluminal coronary angioplasty (PTCA) and stent positioning in 2007, and with an aortocoronary bypass operation and biological aortic valve replacement for bicuspid aortic valve in 2008. In addition, patient history was notable for an urothelial carcinoma of the bladder with radical cystectomy 2 years prior to the actual hospitalization. A cardioembolic origin of the cerebral ischemic was suspected, but atrial fibrillation could not be confirmed during repeated cardiac evaluations. Aspirin was replaced by the direct oral anticoagulant rivaroxaban. In the following 2 months, the patient had two recurrent episodes of myocardial ischemia: the first involving a thrombotic occlusion of the right coronary artery (RCA) and the second due to a reocclusion of the same vessel despite adequate triple antithrombotic therapy with aspirin 100 mg, clopidogrel 75 mg, and rivaroxaban 20 mg.

A low platelet count was already noted at the time of these investigations (95 × 10^9^/L), further decreasing down to 63 × 10^9^/L at the time of the second discharge.

A comprehensive hemato-oncological workup was carried out to investigate the persistent thrombocytopenia and spontaneous cutaneous bleeding events of World Health Organization (WHO) grade 2 while on antithrombotic triple therapy. Comprehensive laboratory studies revealed moderate thrombocytopenia of 52 × 10^9^/L, with the other two lines preserved. No schistocytes or dysplastic elements were observed in the peripheral blood smear. Furthermore, coagulation studies revealed markedly reduced fibrinogen levels (0.5 g/L; normal range 1.7–4.5 g/L), elevated D-dimer levels of 6.97 mg/L (normal range < 0–5 mg/L), a reduced Quick value of 59% (normal range 70–100%), and a shortened activated partial prothrombin time (aPTT) of 21 seconds (normal range 25–37 seconds), but no relevant rivaroxaban activity (39 ng/mL). Vitamin K, haptoglobin, direct Coombs test, careening for anti-PF4 antibodies, and ADAMTS13 activity were within the normal range. The clinical presentation and the laboratory results revealed a picture compatible with overt DIC; consequently, the patient underwent a neoplasia screening. A bone marrow biopsy showed no signs of dysplasia or pathological lymphoid and plasmacellular infiltrates. Karyotype analysis confirmed a normal male karyotype. However, next-generation sequencing showed mutations in *TET2* and *EZH2*, compatible with a diagnosis of clonal hematopoiesis of indeterminate potential (CHIP), which, despite its greater risk for hematological malignancies, has been shown to increase mortality from nonhematological cancers and cardiovascular disease [9], could not alone explain the repeated cardiovascular events experienced by our patient and the coagulation activation.

**Fig. 1:**
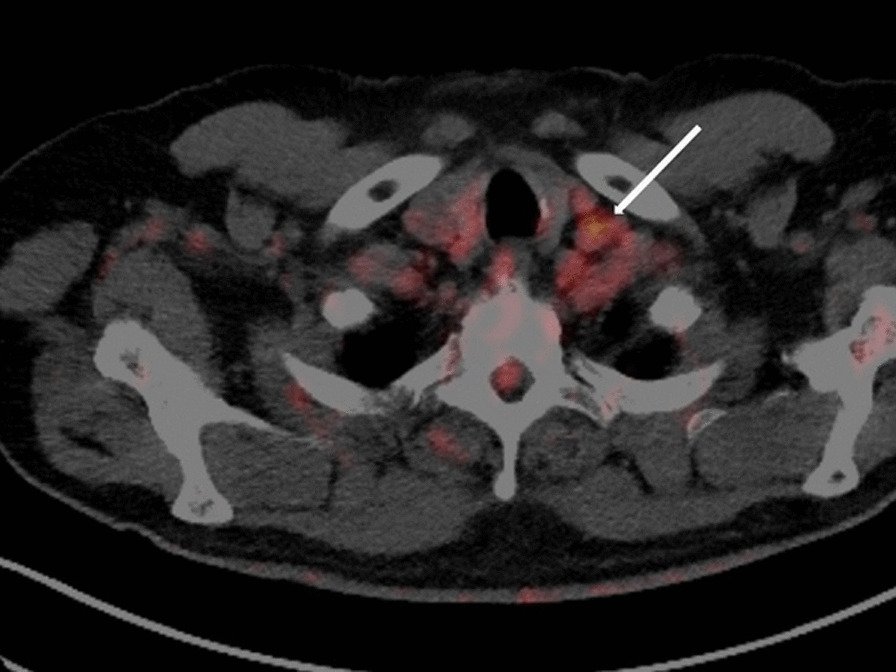
FDG-PET scan showing left supraclavicular lymph nodes with moderate metabolic activity [standardized uptake value (SUV) 4.0]

**Fig. 2:**
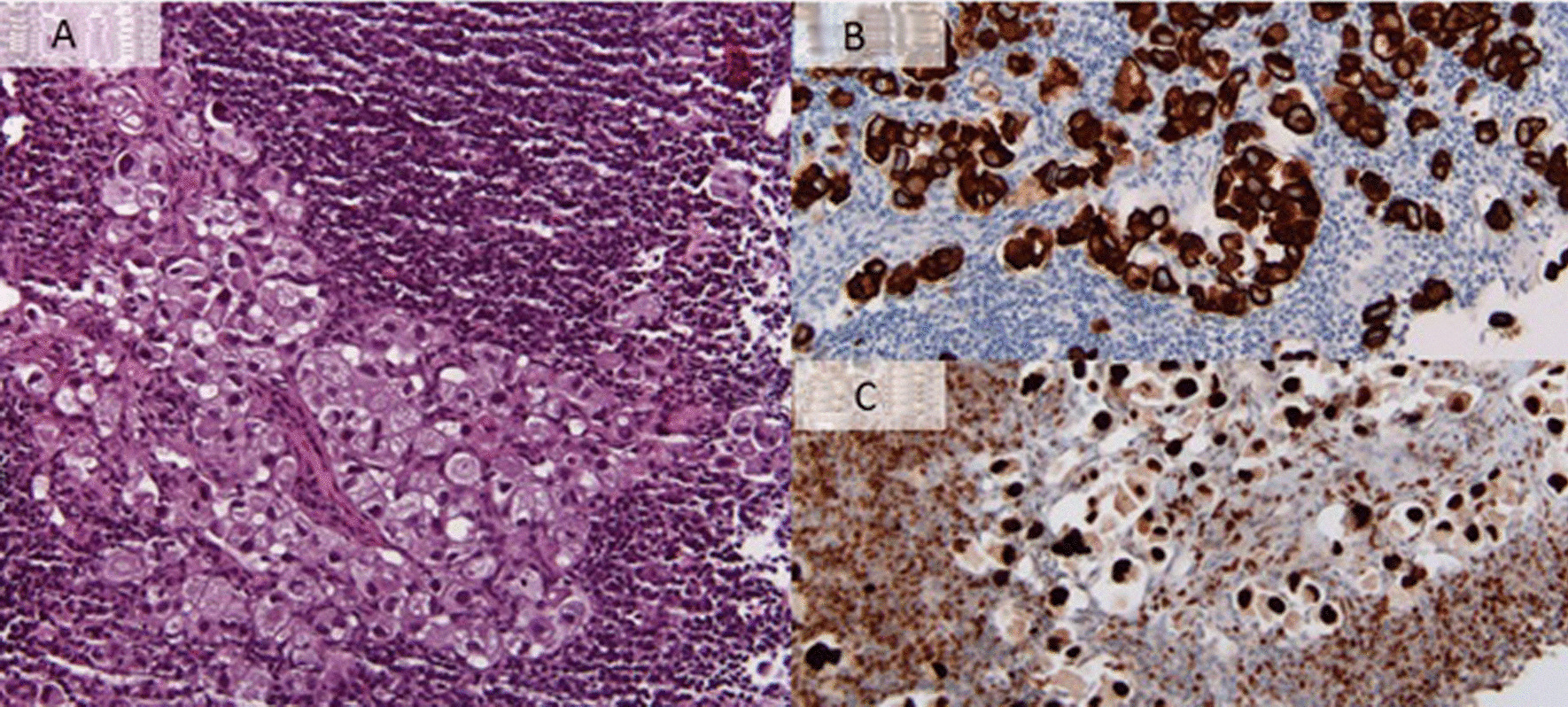
Histological sample from a supraclavicular lymph node. **A** Hematoxylin–eosin staining showing a diffuse infiltration of relatively undifferentiated cells with significant cytologic atypia, hyperchromatic nuclei, and vacuolated cytoplasm. These cells stain positive for pan-cytokeratin (**B**) and GATA3 (**C**)

**Fig. 3:**
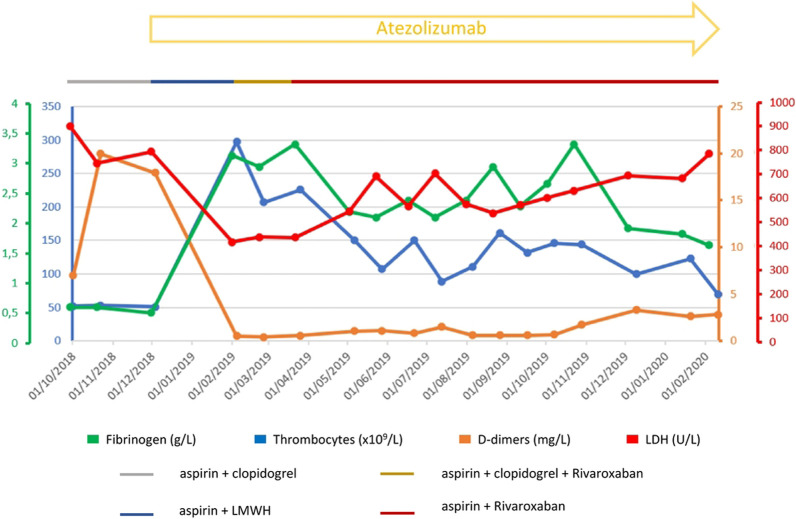
Graphical representation of fibrinogen, thrombocyte count, D-dimers, and lactate dehydrogenase (LDH) values during treatment. Straight lines in the upper part of the picture highlight ongoing antiplatelet and anticoagulant therapies

. Given the high clinical suspicion of an underlying neoplastic disease or chronic infection responsible for the observed coagulation activation, a fluorine-18-fluorodeoxyglucose positron emission tomography (FDG-PET) was performed, and showed few retroperitoneal and left supraclavicular lymph nodes with moderate metabolic activity (Fig. 1). The biopsy and histological workup resulted in the diagnosis of a poorly differentiated carcinoma of urothelial origin (Fig. 2), that is, a relapse of the known bladder urothelial carcinoma. In our institution, the treatment of choice for a patient with metastatic urothelial cancer would have been combination chemotherapy with gemcitabine and either cisplatin or, alternatively, carboplatin in case of ineligibility for cisplatin. However, owing to concerns regarding the hematotoxicity of standard chemotherapy, and the high risk of bleeding in case of worsening of the thrombocytopenia in a patient in need of antithrombotic prophylaxis, we decided to treat the patient with immunotherapy only. The first administration of atezolizumab, an anti-PD-L1 monoclonal antibody, resulted in an improvement of the chronic DIC with normalization of the platelet count and marked reduction of the D-dimer levels from 15 to 1 mg/L (Fig 3). After the first 3 months of treatment, the patient underwent a restaging through a FDG-PET scan, which showed a good metabolic response, without any sign of dimensional increase or appearance of new lesions. The DIC remained well controlled, giving us the opportunity to switch the antithrombotic therapy from aspirin plus low molecular weight heparin to aspirin plus rivaroxaban 20 mg/day.

No further thrombotic events or bleeding events occurred after treatment initiation. In October 2020, after almost 24 months of anti-PD-L1 treatment, the patient is still receiving atezolizumab and is doing well. The patient himself is very happy with his treatment, since he has had no secondary effects related to the administration and he has not been hospitalized again.

## Discussion

We here report the case of a patient with severe thrombotic complications due to paraneoplastic chronic disseminated intravascular coagulation.

There are many things that can be learned from this case. Firstly, thrombocytopenia can be associated with thrombosis, not only with bleeding. Secondly, a comprehensive coagulation assessment including Quick, aPTT, fibrinogen, and D-dimers in patients with thrombocytopenia is mandatory for making the correct diagnosis. Thirdly, chronic DIC is not a disease but a manifestation of an underlying condition and must prompt a thorough checkup for malignant disease. Metastatic disease of supposedly curatively operated neoplasm can recur in distant and unusual sites, and a histological workup is always required. Fourthly, paraneoplastic DIC is reversible if tumor remission can be achieved, irrespective of the type of cancer treatment that has been applied.

DIC is relatively common in cancer patients. Sallah *et al*. reported the occurrence of DIC in 6.8% of their patients evaluated for malignant solid tumors [10] Nevertheless, there is no consensus on how to approach patients with paraneoplastic DIC, apart from the recommendation to treat the underlying disease. Antithrombotic strategies and recommendations for coagulation factor replacement differ widely between various health care systems and institutions.

In 2015, Thachil *et al*. divided cancer-related DIC into three subgroups: “procoagulant” with a tendency for thrombosis, “hyperfibrinolytic” with a tendency for bleeding, and “subclinical.” They suggested prophylactic anticoagulation with low molecular weight heparin (LMWH) or unfractionated heparin (UFH) in those patients with a “procoagulant” presentation when not contraindicated [11], but the cornerstone of treatment remains the treatment of the underlying condition.

In this particular case, we utilized an anti-PD-L1 monoclonal antibody, atezolizumab. It acts as an inhibitor of the PD-L1 receptor, which is overexpressed in different solid tumors; the PD-L1 receptor in turn interacts with the PD-1 receptor on immune cells, preventing T-cell-mediated immune response [12, 13]**.** By blocking this interaction, atezolizumab activates T cells, eventually leading to antitumor response [14, 15]. Checkpoint inhibitors have an increasing role in the treatment of urothelial carcinoma and are now an established treatment option for patients with platinum-refractory locally advanced or metastatic disease [16, 17]

Little is known about the efficacy of immunotherapy, especially checkpoint inhibitors in the treatment of paraneoplastic DIC. After a comprehensive literature review, we identified only one case report describing a young lady with metastatic melanoma and DIC treated with nivolumab and ipilimumab, in which the patient showed complete resolution of her bicytopenia after two cycles of therapy [18].

Our case highlights the importance of extensive diagnostic workup in case of unexplained thrombocytopenia. Immune checkpoint inhibitors will play an important role in managing solid tumors and their complications, especially in patients with contraindication to standard chemotherapeutics. Further studies are needed to define the role and the potential benefits of this new therapeutic approach.

## Conclusions

Chronic DIC is a life-threatening condition, often quite challenging to diagnose. Its management relies on the treatment of the underlying disease. Our case proposes a different therapeutic approach with immunotherapy, owing to the unsuitability of conventional chemotherapy for our patient.

To the best of our knowledge, this is the second case of chronic DIC due to neoplastic disease treated with immunotherapy.

## Data Availability

Not applicable.

## Abbreviations

ACS: Acute coronary syndrome
APS: Antiphospholipid therapy
CHIP: Clonal hematopoiesis of indeterminate potential
DAPT: Dual antiplatelet therapy
DIC: Disseminated intravascular coagulation
HIT: Heparin-induced thrombocytopenia
ITP: Immune thrombocytopenia
MRI: Magnetic resonance imaging
PNH: Paroxysmal nocturnal hemoglobinuria
FDG-PET: Fluorine-18-fluorodeoxyglucose positron emission tomography
PTCA: Percutaneous transluminal coronary angioplasty
RCA: Right coronary artery
